# Which parameters influence cognitive, psychiatric and long-term seizure outcome in mesial temporal lobe epilepsy after selective amygdalohippocampectomy?

**DOI:** 10.1007/s00415-024-12343-y

**Published:** 2024-04-15

**Authors:** Judith Jud, Harald Stefanits, Ellen Gelpi, Valérie Quinot, Susanne Aull-Watschinger, Thomas Czech, Christian Dorfer, Karl Rössler, Christoph Baumgartner, Gregor Kasprian, Clara Watschinger, Doris Moser, Jonas Brugger, Ekaterina Pataraia

**Affiliations:** 1 Department of Neurology, Medical University of Vienna, Währinger Gürtel 18-20, 1090 Vienna, Austria; 2 Comprehensive Center for Clinical Neurosciences & Mental Health, Medical University of Vienna, Vienna, Austria; 3 Department of Neurosurgery, Medical University of Vienna, Vienna, Austria; 4 Division of Neuropathology and Neurochemistry, Department of Neurology, Medical University of Vienna, Vienna, Austria; 5 Department of Neurology, General Hospital Hietzing With Neurological Center Rosenhügel, Vienna, Austria; 6 Karl Landsteiner Institute of Clinical Epilepsy Research and Cognitive Neurology, Vienna, Austria; 7 Department of Neuroradiology, Medical University of Vienna, Vienna, Austria; 8 Vienna General Hospital, Vienna, Austria; 9 Institute of Medical Statistics, Center for Medical Data Science, Medical University of Vienna, Vienna, Austria

**Keywords:** Mesial temporal lobe epilepsy, Hippocampal sclerosis, Selective amygdalohippocampectomy, Neuropsychological outcome, Postoperative seizure outcome

## Abstract

**Background:**

We aimed to analyze potentially prognostic factors which could have influence on postoperative seizure, neuropsychological and psychiatric outcome in a cohort of patients with mesial temporal lobe epilepsy (MTLE) due to hippocampal sclerosis (HS) after selective amygdalohippocampectomy (SAHE) via transsylvian approach.

**Methods:**

Clinical variables of 171 patients with drug-resistant MTLE with HS (88 females) who underwent SAHE between 1994 and 2019 were evaluated using univariable and multivariable logistic regression models, to investigate which of the explanatory parameters can best predict the outcome.

**Results:**

At the last available follow-up visit 12.3 ± 6.3 years after surgery 114 patients (67.9%) were seizure-free. Left hemispheric MTLE was associated with worse postoperative seizure outcome at first year after surgery (OR = 0.54, *p* = 0.01), female sex—with seizure recurrence at years 2 (OR = 0.52, *p* = 0.01) and 5 (OR = 0.53, *p* = 0.025) and higher number of preoperative antiseizure medication trials—with seizure recurrence at year 2 (OR = 0.77, *p* = 0.0064), whereas patients without history of traumatic brain injury had better postoperative seizure outcome at first year (OR = 2.08, *p* = 0.0091). All predictors lost their predictive value in long-term course. HS types had no prognostic influence on outcome. Patients operated on right side performed better in verbal memory compared to left (VLMT 1-5 *p* < 0.001, VLMT 7 *p* = 0.001). Depression occurred less frequently in seizure-free patients compared to non-seizure-free patients (BDI-II Z = − 2.341, *p* = 0.019).

**Conclusions:**

SAHE gives an improved chance of achieving good postoperative seizure, psychiatric and neuropsychological outcome in patients with in MTLE due to HS. Predictors of short-term outcome don’t predict long-term outcome.

**Supplementary Information:**

The online version contains supplementary material available at 10.1007/s00415-024-12343-y.

## Introduction

Mesial temporal lobe epilepsy (MTLE) with hippocampal sclerosis (HS) is the most common focal epilepsy syndrome, which is characterized by drug-resistant seizures [1, 2].

The benefit of surgical treatment compared to medical therapies has been demonstrated in several series [3] revealing a reduction of seizure frequency in approximately two thirds of MTLE patients with HS [2]. Postoperative clinical course is variable and mechanisms of occurrence of postoperative seizures are not always clear. While there is still debate if seizure outcome differs depending on surgical approach, selective resections resulted in better neuropsychological performance [4, 5]. Several potential factors have prognostic impact on seizure outcome. Short duration of epilepsy [2, 6], younger age at surgery [7], low preoperative seizure frequency [8], absence of bilateral tonic–clonic seizures (BTCS) [7] or presence of strictly ipsilateral temporal interictal epileptiform discharges (IED) [7, 8] are associated with better postoperative seizure control. Older age at surgery [6], bitemporal IEDs, bitemporal asynchrony in ictal scalp electroencephalography (EEG) [9], high preoperative seizure frequency [6], BTCS [6, 10] or status epilepticus in history [10] correlate with worse postoperative outcome. Male gender and early onset of seizures are reported to predict good postoperative outcome by some investigators [8], whereas others found controversial results [11].

Based on consensus classification system of three HS types which was recently introduced by the International League against Epilepsy (ILAE) [12], numerous authors investigated prognostic value of different patterns of cell loss in subfields of hippocampal formation on seizure outcome. HS type 1 was found to be associated with better postoperative outcome [12–14], although no significant differences between HS types 1 or 2 and postoperative seizure outcome was found in other studies [15, 16].

In this study, we aimed to analyze potential factors which could have prognostic impact on postoperative neuropsychological, psychiatric and seizure outcome in a very homogeneous cohort of 171 patients with MTLE due to HS who underwent selective amygdalohippocampectomy (SAHE) via transsylvian approach and were followed for up to 25 years.

## Methods

### Patients

We retrospectively analyzed data on postoperative outcome of all adult patients with drug-resistant MTLE who underwent an extensive presurgical evaluation and subsequent SAHE at the Vienna Epilepsy Surgery Program of the Medical University of Vienna, Austria between January 1994 and June 2019. Only patients with unilateral MTLE with HS with a minimum of 12 months follow-up after surgery were included in the study. The study was approved by local ethics committee (EK 2071/2017 and EK 1137/2014).

### Presurgical evaluation

Presurgical work-up comprised clinical history, neurological and cognitive evaluation, prolonged video-electroencephalography (EEG)-monitoring, high-resolution magnetic resonance imaging (MRI), neuropsychological testing including psychiatric evaluation, assessment of language functions with Wada-test or functional MRI, visual field examination and recently, 18F-Fluorodeoxyglucose positron emission tomography.

Prolonged video-EEG monitoring was performed for an average of 5 days (3–8 days) using a 32-channel video-EEG system with electrodes placed according to the International 10–20 System with additional bilaterally placed true anterior temporal electrodes and sphenoidal electrodes.

Spike frequency and location of IEDs were evaluated by visual analysis. Clinical semiology was evaluated with respect to localizing and lateralizing signs to predict the seizure onset zone [17]. Ictal EEG patterns were defined by morphology, localization and temporal evolution [18].

High-resolution MRIs were performed with 1.5-Tesla (Gyroscan ACS-NT, Philips Medical Systems) and starting 2006 with a 3-Tesla machine (Siemens Magnetom Trio, Siemens Medical Systems). For detection of epileptogenic lesions the MRI protocol recommended by ILAE was applied [19].

Standardized neuropsychological testing included assessment of global intellectual function (short form of HAWIE), verbal and nonverbal episodic memory (Verbaler Lern- und Merkfähigkeitstest (VLMT), a German version of Rey Verbal Learning test) and the Diagnosticum für Cerebralschädigung, revised version (DCS-R), phonematic and semantic fluency, attention (d2 test), executive functions (subset of LPS-7 test and labyrinth test), visuo-constructive functions (subtest of HAWIE-R) and for evaluation of psychiatric comorbidities Beck Depression Inventory (BDI-II) (see Supplemental references for neuropsychological tests).

### Epilepsy surgery

Decision regarding surgical approach was made individually after case discussion in the multidisciplinary epilepsy conference including epileptologists, neurosurgeons, neuroradiologists and neuropsychologists. All surgeries were performed by one neurosurgeon (T.C.) consisting of SAHE via transsylvian approach [20, 21].

### Histopathological examination

Resected hippocampal specimens were dissected orthogonally to the longitudinal axis into three- to five-millimeter-thick slices. For tissue fixation slices were immersed overnight in 4% buffered formaldehyde solution, and routinely embedded into liquid paraffin. Paraffin block was cut at three- to five-micrometer-thick sections with a microtome and routinely stained with hematoxylin and eosin, and luxol-fast-blue/Kernechtrot (Klüver-Barrera).

Immunohistochemistry was performed on selected sections, i.e., mid part of the hippocampus, applying at least the following primary antibodies: Anti-NeuN (neuronal nuclear antigen; MAB377, clone A60, 1:100, EMD Millipore, Darmstadt, Germany) and anti-GFAP (glial fibrillary acidic protein; 0761, clone 6 F2, 1:500, Dako, Glostrup, Denmark). The Envision kit (Dako) was used as detection system and diaminobenzidine for visualization of antigen–antibody-reaction.

Resected tissue was reevaluated regarding to completeness of all regions and reclassified according to new ILAE Classification [12] separately by two of the authors experienced in assessing HS (V.Q. and H.S.) and supervised by board certified neuropathologist (E.G.), who were blinded to the clinical information of patients. Evaluation of neuronal cell loss was based on a semi-quantitative visual evaluation. If the classification diverged between the assessors the slides were reviewed together to reach consensus.

### Postoperative outcome assessment

All patients were regularly followed at 1, 2 and 5 years and subsequently every 5 years (up to 25 years) after surgery. Postsurgical data were collected during outpatient visits and included assessment of seizure frequency according to ILAE Classification [22], current anti-seizure medication (ASM) regimen, neurological examination, neuropsychological testing and scalp EEG. Most recent retrieval of postoperative seizure outcome was December 31, 2021.

### Statistical analysis

We evaluated following variables: sex, handedness, initial precipitating injuries (IPIs) (perinatal insult, febrile convulsions, traumatic brain injury, meningitis/encephalitis), preoperative seizure frequency, number of ASM, history of BTCS, results of video-EEG-monitoring (IEDs, ictal EEG patterns and clinical seizure semiology), side of resection, age at epilepsy onset, duration of epilepsy and age at surgery.

For each year, we defined binary outcome as “seizure-free” (SF) if the outcome was Class 1a (completely SF after surgery) or Class 1 (SF at least 12 months prior to last follow-up assessment) and as “not seizure-free” (NSF) (Class 2–6). We defined univariable logistic regression models with binary outcome as the dependent and all considered clinical and morphological parameters as the explanatory variables for each outcome year separately. We calculated Odds-Ratios and Wald-test p-values for each potential explanatory parameter. Additionally, we computed likelihood-ratio tests for each explanatory parameter to test for an overall significant association with the examined outcome. If the explanatory parameter is categorical, this is equivalent to performing a Chi-square test for independence. We then fitted multivariable logistic regression models using the parameters that yielded a p-value < 0.1 in the likelihood-ratio tests. We again computed Odds-Ratios and p-values of all parameters in the adjusted models. For the entire analysis, we used Firth’s bias reduction method since some combination of variables allow for perfect separation. No correction for multiple testing was performed, therefore, all p-values are of descriptive, hypothesis-generating character. All calculations were performed using R, version 3.6.1 or higher. We used the package “logistf” to compute the likelihood-penalized logistic regression models.

Statistical program package SPSS (version 22.0, Chicago, IL, USA) was used for neuropsychological data analysis. Scores of global intellectual functions, visual and verbal short- and long-term memory, semantic and phonematic fluency, attention, executive functions and depression scale preoperatively and 12 months after surgery were evaluated by means of analysis of variance (ANOVA). Independent variables were side of resection (left/right), seizure outcome (SF/NSF) and HS type (HS type 1/HS type 2). Throughout analysis, we defined the significance level as *α* = 0.05. However, no correction for multiple testing was applied, therefore all *p*-values are of descriptive character. Descriptive summary statistics of discrete variables were calculated as counts (relative frequency) and as mean ± standard deviation in case of continuous parameters.

## Results

### Patient population

One hundred-and-seventy-one patients with drug-resistant MTLE with HS (88 females) with a mean age of 36.9 ± 10.1 years (range 16.4–62 years) who underwent SAHE at our center were included in the study. Patient characteristics are summarized in Table 1.

**Table 1:** Clinical characteristics

	Number of patients (%)
Patients	171 (100)
Sex (female/male)	88 (51.5)/83 (48.5)
Handedness (right/left/bimanual)	155 (90.1)/14 (8.2)/2 (1.2)
IPI	
Febrile convulsions	44 (25.7)
Perinatal insult	14 (8.2)
Meningitis/Encephalitis	48 (28.1)
Traumatic brain injury	36 (21.1)
High seizure frequency preoperatively^a^	112 (65.5)
Number of ASM	4.8 ± 2.6 (1.0–15)
Results of Video-EEG monitoring^b^	
Clinical seizure semiology	162 (94.7)
Temporal ictal EEG pattern	160 (92.6)
Unitemporal IEDs	159 (92.0)
History of BTCS	135 (78.9)
Dystonic posturing of hand	46 (26.9)
Postictal symptoms	
Confusion/Aphasia	102 (59.6)/51 (29.8)
Psychosis	9 (5.3)
Paresis	16 (9.4)
Side of resection (left/right)	95 (55.6)/76 (44.4)
Histopathology	
HS type	96 (56.2)
Type 1	75 (78.0)
Type 2	18 (18.9)
Type 3	1 (1.0)
No HS	2 (2.1)
HS-nos	50 (29.2)
Specimen not available	25 (14.6)
	Mean ± SD (Range)
Age at epilepsy onset	12.0 ± 10.5 (0.1–47.0)
Duration of epilepsy	24.8 ± 12.8 (1.3–61.0)
Age at surgery	36.9 ± 10.1 (16.4–62.0)
Duration of postoperative follow-up	12.3 ± 6.3 (1.0–25.6)

IPI, initial precipitating injury; ^a^, more than one seizure per week; ^b^, ipsilateral to side of resection; ASM, anti-seizure medication; EEG, electroencephalography; IEDs, interictal epileptiform discharges; BTCS, bilateral tonic–clonic seizures; HS, hippocampal sclerosis; HS-nos, hippocampal sclerosis not otherwise specified (subtyping not possible due to tissue fragmentation and/or lack of representation of all CA sectors)

At least one risk factor was reported in 116 patients (67.8%): 28 patients (16.3%) had a history of more than one IPI (25 patients had 2 IPIs and 3 patient 3 IPIs), 44 patients (25.7%) had febrile seizures, 14 patients (8.2%) had a perinatal insult, 48 patients (28.1%) suffered from meningitis or encephalitis in early childhood and 36 patients (21.1%) had traumatic brain injuries before seizure onset.

Mean age at epilepsy onset was 12.0 ± 10.5 years (range 0.1–47 years). 112 patients (65.5%) had a high seizure frequency (> 1 seizure per week). 135 patients (78.9%) had a preoperative history of BTCS. The mean preoperative ASM trials were 4.8 ± 2.6 (range 1–15 ASM). The MRI showed unilateral HS in 166 patients (97.1%).

Clinical lateralizing signs were concordant to side of resection in 162 patients (94.7%), ictal EEG-patterns—in 160 patients (92.6%) and IEDs—in 159 patients (92%).

Eight patients (4.7%) underwent invasive recordings either due to discordant video-EEG-results or missing signs of HS on MRI. Mean duration of epilepsy prior to surgery was 24.8 ± 12.8 years (range 1.3–61 years), mean age at surgery—36.9 ± 10.1 years (range 15.4–62 years). Surgery was performed on the left temporal lobe in 95 patients (55.6%). Surgical complications with temporary morbidity were seen in 13 patients (7.6%): Ten patients (5.8%) developed postoperative hygroma, of which two required drainages through a burr hole, one patient needed evacuation of a hematoma in the resection cavity on the second postoperative day, one patient had an infarct in the territory of the lateral posterior choroidal artery and two patients had temporary oculomotor nerve palsy. None of these patients had long-term neurological sequelae.

### Postoperative outcome

Postoperative seizure outcome for at least 12 months was available for 168 (98.2%) patients.

**Fig. 1:**
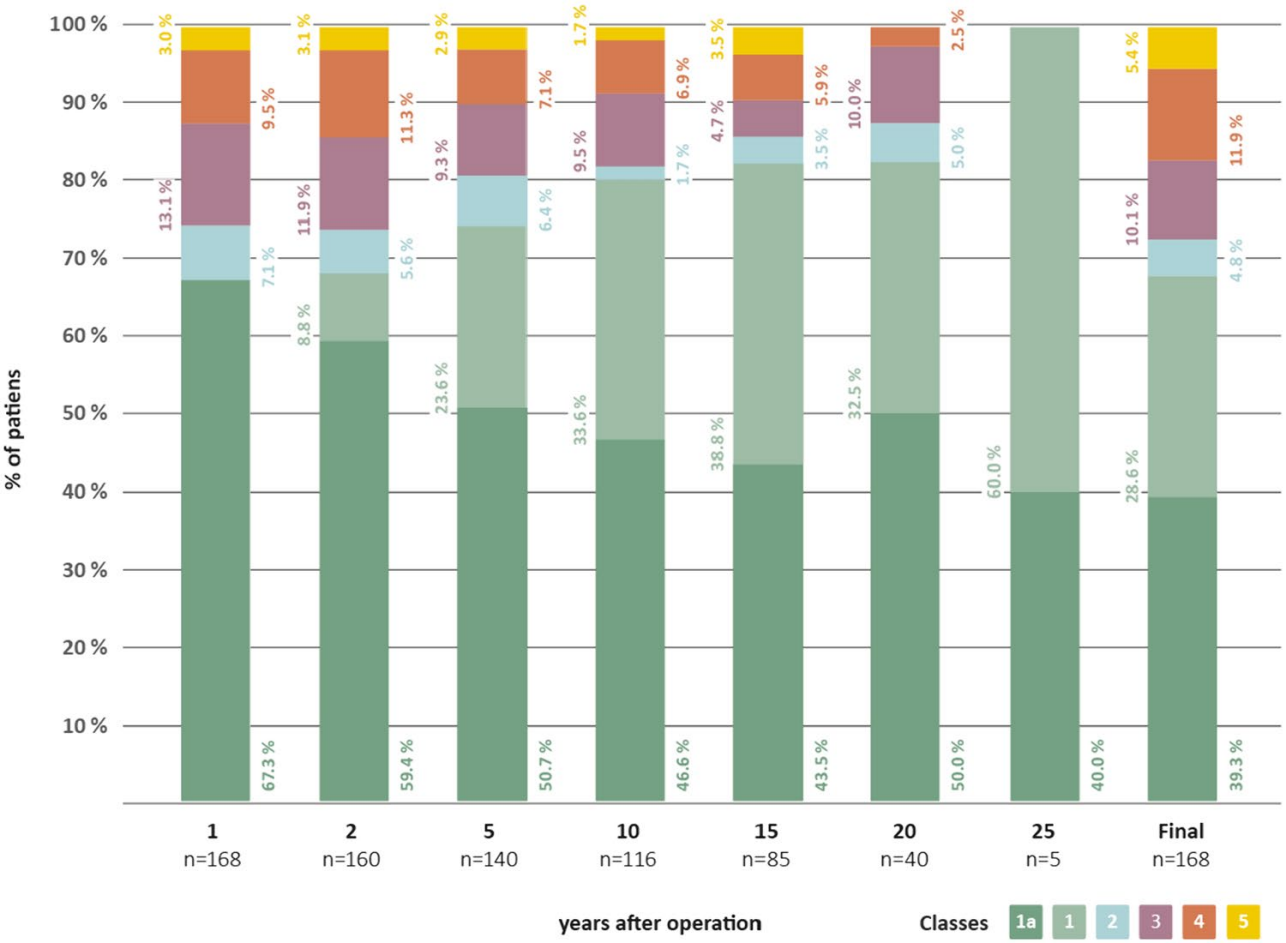
Long-term postoperative seizure outcome which was classified according to ILAE classification system [22]. *X*-axis: years (1, 2, 5, 10, 15, 20, 25 year(s), and last available follow-up visit, respectively) after surgery, *y*-axis: relative frequency of patients

At the last available follow-up 12.3 ± 6.3 years after surgery (range 1–25.6 years) 114 patients (67.9%) were seizure-free (ILAE Class 1a and Class 1): of these, 66 patients (39.3%) were completely seizure-free since surgery (ILAE Class 1a) and 48 patients (28.6%) had no seizures at least 12 months before last evaluation. Additional 54 patients (32.1%) also benefitted from the surgery: Eight patients (4.8%) had only auras (ILAE Class 2), 17 patients (10.1%) had 1–3 seizure-days per year (ILAE Class 3) and 20 patients (11.9%) achieved ILAE Class 4 (up to 50% reduction of baseline seizure-days). Only 9 patients (5.4%) had no improvement of seizure frequency (ILAE Class 5). There were no patients with appreciable worsening of seizures (ILAE Class 6) (Fig. 1).

Forty-three seizure-free patients (25.6%) were off-medication at the last available follow-up.

Over the course of the years, 15 patients (8.8%) died (average 10.4 ± 6.1 years after surgery; range 1–20.9 years). Six patients died due to tumors (one patient with colorectal cancer, four patients with bronchial cancer, one patient with multiple myeloma and two patients with glioblastoma). Two patients (both seizure-free after surgery) committed suicide due to severe depression. One patient died in status epilepticus, and the reason of the death of four patients, who were not seizure-free after surgery, could not be resolved (we suspect that one patient drowned during a seizure and three patients died in seizure, where sudden unexpected death in epilepsy patients (SUDEP) were suspected, although no autopsy was performed).

Seven patients (4.2%) were lost of follow-up.

Twelve patients (7.1%) underwent reoperations with a mean of 4.9 ± 3.8 years (range 1–12.9 years) after failure of the initial surgery and were followed for 13.5 ± 7.0 years (1.3–21.3 years). At the last available follow-up eight of reoperated patients were seizure free (three patients with ILAE class 1a and five patients with ILAE class 1) and four patients were not seizure free*.*

### Assessment of predictors for seizure outcome

We applied univariable logistic regression models and computed likelihood-ratio tests for each explanatory parameter to test for an overall significant association with the examined outcome (Supplemental Table S1). We then fitted multivariable logistic regression models to investigate which of the explanatory parameters can predict the outcome best. Left hemispheric MTLE was associated with worse postoperative seizure outcome at first year after surgery (OR = 0.54, *p* = 0.01). Higher number of preoperative ASM trials at year 2 (OR = 0.77, *p* = 0.0064) and female sex were associated with seizure recurrence at years 2 (OR = 0.52, *p* = 0.01) and 5 (OR = 0.53, *p* = 0.025) after surgery. Patients without history of traumatic brain injury had better postoperative seizure outcome at first year after surgery (OR = 2.08, *p* = 0.0091) (Supplemental Fig. S1).

### Histopathological tissue evaluation

For evaluation of the impact of different HS types on postoperative seizure outcome only patients with representation of all hippocampal sectors on histology were included into subsequent calculation. 25 patients (14.6%), where hippocampal specimens were not available and hippocampal specimens of 50 patients (29.2%), which were classified as HS-nos (HS, not otherwise specified) due to tissue fragmentation and incompleteness of hippocampal sectors (Supplemental Fig. S2) were also excluded.

Final dataset comprised 96 cases with all identified and represented hippocampal sectors.

The tissue samples of 75 patients (78%) were assigned to HS type 1, 18 patients (18.9%) to HS type 2, one patient (1%) for HS type 3 and two patients (2.1%) had no-HS according to ILAE classification. The last three samples (with HS type 3 and no-HS) were excluded from further statistical analysis due to small number of cases. HS type was not significantly associated with seizure outcome at any time of postoperative assessment according to the univariable models.

### Neuropsychological and psychiatric outcome

Over the years, tests were adapted and some new tests were introduced. Only the data of 63 patients with complete pre- and postoperative test batteries were analyzed (Supplemental Table S2).

Preoperative testing showed unaffected global intelligence in 31 patients (57.4%).

Postoperative testing showed increased overall global intellectual functioning (HAWIE-R *p* = 0.019), improvement in attention (D2-TS-E *p* = 0.003), and slight improvement in word fluency performance (phonematic word fluency *p* < 0.001, semantic word fluency *p* = 0.018).

Overall, there was a significant decline in nonverbal memory functions (DCS II 1-5 p < 0.001, DCS 6 *p* < 0.001) as well as decline in verbal memory and learning functions (VLMT 1-5 *p* = 0.008, VLMT 7 *p* = 0.001), although patients operated on the right side performed better in verbal memory than patients operated on the left side (VLMT 1-5 *p* < 0.001, VLMT 7 *p* = 0.001) (Supplemental Table S3). There was no significant difference in memory functions between seizure-free and not seizure-free patients.

Depression scale revealed depression in 16 patients (25.4%) preoperatively. Postoperative psychiatric evaluation showed significant improvement of depression in all patients (BDI-II *p* < 0.001), whereas seizure-free patients scored lower compared to not seizure-free patients (BDI-II *Z* = − 2.341, *p* = 0.019).

There was no significant difference in memory function and depressive symptoms between patients with HS type 1 and HS type 2 neither in preoperative nor in postoperative testing.

## Discussion

We assessed long-term postoperative seizure outcome in a very homogeneous cohort of patients with MTLE due to HS, who underwent SAHE via transsylvian approach at a single level-4 epilepsy center. Overall, 67.9% of patients were seizure-free at the last available follow-up 12.3 ± 6.3 years after surgery (ILAE Class 1a and Class 1).

Our results are mostly consistent with previously published data on seizure outcome after SAHE [5], including our own experience [20], with 34–93% Engel Class 1 [23] seizure outcome at the last available follow-up. Reasons for variability in outcome demonstrated by different studies may result from selection of the initial patient population with predominantly lower number of patients [20, 24] and the learning curve of surgeons, different surgical approaches (transsylvian [2, 20, 25] vs. transcortical [16, 24] route), surgical complications, pharmacological factors (different ASM regimen postoperatively), psychosocial factors (i.e., driving permit), or finally, from natural course of disease (i.e., psychiatric comorbidities, SUDEP [26]). Nevertheless, all these studies emphasize the effectiveness of surgical treatment.

Proportion of completely seizure-free patients since surgery declined over time from 67% at first postoperative year to 40% 25 years after surgery, resulting in a 39.3% of complete seizure-freedom (ILAE Class 1a) at the last available follow-up.

Recognition of prognostic factors is very important for identifying patients’ risk of postoperative seizure recurrence. We were able to identify several predictors for surgical outcome: left hemispheric MTLE was associated with worse postoperative seizure outcome at first year after surgery, female sex was associated with seizure recurrence at years two and five, and higher number of preoperative ASM trials at year two after surgery. Patients without history of traumatic brain injury had better postoperative seizure outcome at first year after surgery. All these predictors lost their predictive value over the years.

The most common histopathological finding in our patients was HS ILAE type 1. Less frequent subtypes of HS (HS ILAE type 2 and 3 and no HS) were observed in 12.2% (21.9%) of cases, which was similar to recent studies [16, 27, 28], but differed from earlier reports [13, 29]. These differences could be related to different patient selection criteria, surgical procedure and/or stringent application of histopathological criteria [16, 30].

The prognostic value of HS patterns regarding postoperative seizure outcome is also different among studies. Whereas improved postoperative seizure outcome with seizure-freedom of 61–85% was demonstrated for patients with HS ILAE type 1 in some studies [13, 31, 32], no association between HS patterns and postoperative seizure outcome was reported in other series [15, 16, 27]. These latter results are similar to ours, since we could not find a prognostic value of HS types 1 or 2 on seizure outcome.

Epilepsy surgery is considered as treatment of choice for drug resistant MTLE with HS and a safe procedure, although verbal memory worsening is expected, especially if surgery is performed on the dominant hemisphere [4, 33]. In our population, patients who underwent surgery on the right (non-dominant) hemisphere performed better in verbal memory compared to patients after left-sided resection. These results are similar to previously published studies [4, 14, 16, 33, 34]. Global intelligence was not affected in 57.4% of our patients preoperatively. Postoperative neuropsychological assessment showed increased overall global intellectual functioning, improvement in attention, and slight improvement in word fluency performance.

Prognostic value of different HS ILAE types on memory dysfunction is controversially discussed in the literature. Whereas some authors suggested no significant correlation between HS types and pre- or postoperative memory dysfunction [14], others reported better preoperative verbal memory performance in patients with left-sided HS ILAE type 2 and no HS compared to patients with left-sided HS ILAE type 1 or 3 [31, 33], or no significant difference in cognitive performance preoperatively regardless of side of resection or cognitive decline between patients with HS ILAE type 1 or 2 [34]. We could not identify any difference in memory function between patients with different HS ILAE types neither in preoperative nor in postoperative testing.

A significant memory decline during long-term follow-up in medically treated patients has been demonstrated previously [35, 36]. Seizure-freedom in surgically treated patients can positively influence memory performance during long-term follow-up [37] and poor postoperative seizure control is assumed as a potential risk factor for postoperative memory decline [36].

No significant link of seizure outcome on memory functions was observed in our patient population. This is in line with previous reports [38, 39].

In one of three people with epilepsy a lifetime history of psychiatric disease is reported, identifying depression as one of the most frequent psychiatric comorbidity [40], affecting 23% of people with epilepsy, with an 2.7-fold increased overall risk compared to general population. Similarly, depression was reported preoperatively in a quarter of patients in our cohort. Epilepsy surgery seems to have a positive influence on psychiatric outcome, showing significant improvement of depression postoperatively [16]. Similarly, in our patient population, depression occurred less frequently in seizure-free patients compared to not seizure-free patients.

Our study has some limitations: (1) analysis of histopathological tissues was limited by the small number of patients within each subgroup, since, despite having HS, histological sub-classification was not possible in all cases due to tissue fragmentation or incomplete representation of all CA sectors, which might partly explain the lack of association between different HS types and surgical outcome. (2) Although all patients underwent neuropsychological assessment pre- and postoperatively, tests were adapted over years and statistical analysis of neuropsychological tests in the present study was done only in cases, where complete and comparable pre- and postoperative test batteries were present. 3) Limited comparability of the results with other series due to the use of distinct outcome assessment systems: we applied the very strict ILAE seizure outcome classification [22], whereas the less strict Engel’s classification system is still used in the majority of studies. And 4) the retrospective nature of the study.

Nevertheless, in this to our knowledge largest homogeneous series of patients with MTLE and HS who underwent SAHE by transsylvian approach in an epilepsy center and were followed for 12.3 ± 6.3 years, we could demonstrate a favorable outcome with 67.9% seizure-freedom at the last available follow-up. It is important to underscore, that surgery positively influences psychiatric and neuropsychological outcome, but also, that all predictors for the first years lost their predictive value in the course of time.

### Supplementary Information

Below is the link to the electronic supplementary material.

Supplementary file1 (DOCX 1845 kb)

## Data Availability

All data relevant to the study are included in the article or uploaded as supplementary information.
