# Supplementary material for: Which parameters influence cognitive, psychiatric and long-term seizure outcome in mesial temporal lobe epilepsy after selective amygdalohippocampectomy?
Source: J Neurol. 2024 Apr 15;271(7):4249–57. doi: 10.1007/s00415-024-12343-y (PMC11233333; doi:10.1007/s00415-024-12343-y)
Supplement: Supplementary file 1 — Supplementary file1 (DOCX 1845 kb) [file 415_2024_12343_MOESM1_ESM.docx]

**Supplemental INFORMATION**

| **Supplemental Table S1** Univariable models of postoperative seizure outcome at year(s) 1, 2, 5, 10, 15 and 20 after surgery | | | | | | |
| --- | --- | --- | --- | --- | --- | --- |
|  | 1 year | 2 years | 5 years | 10 years | 15 years | 20 years |
|  | n=168 | n=160 | n=140 | n=116 | n=85 | n=40 |
| **Variables** |  | | | | | |
| Female sex | OR=0.30, p=0.4069 | **OR=0.15, p=0.0072** | **OR=0.16, p=0.0252** | OR=0.29, p=0.4843 | OR=0.48, p=0.4203 | OR=0.16, p=0.2619 |
| Handedness  Left/Bimanual | p=0.3198 OR=7.08/OR=2.80 | p=0.4096 OR=1.83/OR=1.51 | p=0.4923 OR=4.62/OR=2.92 | p=0.5373 OR=3.40/OR=2.43 | p=0.9475  OR=2.37/-- | p=0.6901  OR=1.84/-- |
| IPI  Perinatal insult  Febrile convulsions  Meningitis/Encephalitis  Traumatic brain injury | OR=1.90, p=0.2950  OR=3.53, p=0.3635  OR=3.10, p=0.6096  **OR=1.68, p=0.0149** | OR=2.19, p=0.6003  OR=2.54, p=0.8138  OR=2.90, p=0.7863  OR=4.06, p=0.2548 | OR=2.07, p=0.5381  OR=3.09, p=0.6549  OR=4.06, p=0.2597  OR=2.63, p=0.9777 | OR=3.77, p=0.4671  OR=3.88, p=0.3622  OR=7.44, p=0.0647  OR=2.03, p=0.4125 | OR=2.67, p=0.8029  OR=6.78, p=0.1337  OR=5.12, p=0.2236  OR=2.01, p=0.4893 | OR=6.02, p=0.2016  OR=3.67, p=0.5853  OR=2.96, p=0.8053  OR=2.77, p=0.8014 |
| High^1^ seizure frequency preoperatively | OR=0.22, p=0.0802 | OR=0.21, p=0.0800 | OR=0.25, p=0.2366 | OR=0.37, p=0.9446 | OR=0.36, p=0.8812 | OR=0.23, p=0.1798 |
| History of BTCS | OR=0.34, p=0.7519 | OR=0.41, p=0.7303 | OR=0.38, p=0.9894 | OR=0.43, p=0.6566 | OR=0.43, p=0.7159 | p=0.54, p=0.4253 |
| Video-EEG-monitoring^2^  Interictal IEDs | OR=2.08, p=0.5017 | OR=2.30, p=0.7432 | OR=1.73, p=0.3118 | OR=3.33, p=0.5706 | OR=4.05, p=0.2757 | OR=3.10, p=0.3736 |
| Ictal EEG pattern | OR=2.83, p=0.8480 | OR=2.48, p=0.9233 | OR=1.81, p=0.4770 | OR=2.59, p=0.8387 | OR=4.05, p=0.2757 | -- |
| Clinical seizure semiology | OR=1.97, p=0.4521 | OR=1.77, p=0.2751 | OR=1.73, p=0.3118 | OR=2.94, p=0.6937 | OR=1.37, p=0.1281 | OR=2.16, p=0.5322 |
| No MRI signs of HS | OR=1.81, p=0.4705 | OR=1.78, p=0.4467 | OR=1.60, p=0.4595 | OR=1.44, p=0.3360 | OR=2.12, p=0.5319 | OR=2.16, p=0.5322 |
| Dystonic posturing of hand | OR=2.23, p=0.4033 | OR=4.18, p=0.1837 | OR=3.55, p=0.4242 | OR=4.53, p=0.2423 | OR=2.30, p=0.7550 | OR=4.93, p=0.3632 |
| Postictal symptoms  Confusion | **OR=0.19, p=0.0301** | OR=0.24, p=0.1476 | OR=0.43, p=0.5416 | OR=0.44, p=0.5699 | OR=0.45, p=0.6159 | OR=0.51, p=0.5322 |
| Aphasia | OR=2.03, p=0.1665 | OR=2.35, p=0.5547 | OR=3.50, p=0.4229 | OR=2.18, p=0.5068 | OR=7.27, p=0.1172 | OR=23.07, p=0.0628 |
| Paresis | OR=3.58, p=0.4787 | OR=2.52, p=0.8990 | OR=3.87, p=0.4341 | OR=1.86, p=0.4211 | OR=2.23, p=0.8377 | OR=1.45, p=0.1213 |
| Psychosis | OR=3.81, p=0.4754 | OR=1.63, p=0.1312 | **OR=41.6, p=0.0265** | OR=2.94, p=0.6937 | OR=2.67, p=0.8029 | OR=2.11, p=0.8769 |
| Left-sided resection | **OR=0.18, p=0.0219** | OR=0.22, p=0.0895 | OR=0.41, p=0.6904 | OR=0.36, p=0.9615 | OR=0.35, p=0.8934 | OR=0.52, p=0.4035 |
| HS type  HS type 2  HS type 3  No HS | p=0.3484  OR=1.35  OR=1.57  OR=1.47 | p=0.4494  OR=3.26  --  OR=5.43 | p=0.6503  OR=3.40  --  OR=2.58 | p=0.7044  OR=2.38  --  OR=3.63 | p=0.7531  OR=2.80  --  OR=3.02 | p=0.6597  OR=2.01  --  OR=2.25 |
| Age at seizure onset | OR=1.01, p=0.6463 | OR 1.00, p=0.8060 | OR=1.02, p=0.3831 | OR=1.02, p=0.3564 | OR=1.03, p=0.3855 | OR=0.96, p=0.3553 |
| Age at surgery | OR=1.01, p=0.3920 | OR=0.98, p=0.1758 | OR=1.00, p=0.9528 | OR=0.98, p=0.2820 | OR=0.98, p=0.5107 | OR=0.99, p=0.9023 |
| Preoperative ASM trials | **OR=0.85, p=0.0142** | **OR=0.79, p<0.001** | **OR=0.85, p=0.0281** | OR=0.95, p=0.5242 | OR=0.92, p=0.4842 | OR=1.29, p=0.2726 |
| OR=Odds Ratio; IPI= initial precipitating injury; ^1^=more than one seizure per week; BTCS=bilateral tonic-tonic seizures; ^2^=contralateral to side of lesion; EEG= EEG=electroencephalography; IEDs=interictal epileptiform discharges; HS=hippocampal sclerosis; Note: OR of discrete variables are with regards to the most common factor within the group. | | | | | | |

**Supplemental Table S2** Neuropsychological assessment of pre- and postoperative results

|  | N | Preoperative | Postoperative | T | p-value |
| --- | --- | --- | --- | --- | --- |
| HAWIE-R | 52 | 86.63+17.03 | 89.41+16.86 | -2.427 | **0.019** |
| TMT-A | 63 | 34.87+16.78 | 31.60+15.78 | 2.133 | 0.037 |
| TMT-B | 62 | 93.18+6.82 | 89.39+6.16 | 0.960 | 0.341 |
| D2-TS | 55 | 383.76+14.24 | 412.29+16.09 | -2.748 | **0.008** |
| Phonematic word fluency | 62 | 23.42+10.03 | 28.03+9.95 | -4.932 | **0.000** |
| Semantic word fluency | 61 | 16.49+6.33 | 18.25+6.06 | -2.430 | **0.018** |
| VLMT 1-5 | 63 | 45.81+10.32 | 42.65+12.97 | 2.757 | **0.008** |
| VLMT 7 | 63 | 7.70+4.36 | 6.17+4.21 | 3.658 | **0.001** |
| VLMT RC | 63 | 10.25+5.21 | 8.89+5.71 | 1.752 | 0.085 |
| DCS-II 1-5 | 63 | 20.89+10.88 | 16.63+10.08 | 4.998 | **0.000** |
| DCS 6 | 58 | 5.55+2.83 | 4.26+2.67 | 5.130 | **0.000** |
| DCS RC | 51 | 26.22+3.18 | 25.12+3.40 | 2.618 | **0.012** |
| Visuoconstruction | 61 | 28.25+10.39 | 30.44+11.71 | -0.979 | 0.332 |
| Labyrinth | 51 | 12.54+8.70 | 13.77+6.91 | -1.914 | 0.060 |
| LPS-7 | 62 | 15.26+6.37 | 18.32+8.26 | -3.563 | **0.001** |
| BDI-II | 57 | 9.47+6.70 | 6.21+6.68 | 4.452 | **0.000** |
| HAWIE-R=Hamburg Wechsler Intelligence Test – Revised version; TMT=Trail Making Test; D2=test of attention; VLMT=Verbal Learning and Memory Test; DCS= Learning and Memory Test for detecting memory deficits resulting from acquired brain damage; LPS=Performance Testing System; BDI=Beck Depression Inventory;  Values are demonstrated by means ± standard deviation; VLMT: total learning (Trials 1–5), delayed free recall (Trial 7) and recognition (RC); DCS-II: total learning (Trials 1–5), delayed free recall (Trial 6) and recognition (RC). | | | | | |

**Supplemental Table S3** Neuropsychological assessment of postoperative results on patients operated on left and right temporal lobe

|  | N left | N right | left | right | Z | p-value |
| --- | --- | --- | --- | --- | --- | --- |
| HAWIE-R | 31 | 26 | 91.31+16.67 | 89.08+16.97 | -0.321 | 0.749 |
| TMT-A | 35 | 28 | 30.14+15.15 | 33.43+16.20 | -1.073 | 0.283 |
| TMT-B | 35 | 27 | 87.91+52.67 | 91.30+43.51 | -0.611 | 0.541 |
| D2-TS | 33 | 26 | 409.39+120.19 | 405.19+120.96 | -0.298 | 0.766 |
|  |  |  |  |  |  |  |
| Phonematic word fluency | 35 | 27 | 28.14+10.35 | 27.89+9.60 | -0.313 | 0.755 |
| Semantic word fluency | 35 | 27 | 17.94+6.55 | 18.81+5.39 | -0.441 | 0.659 |
| VLMT 1-5 | 35 | 28 | 37.60+12.85 | 48.96+10.21 | -3.751 | **0.000** |
| VLMT 7 | 35 | 28 | 4.60+3.87 | 8.14+8.14 | -3.182 | **0.001** |
| VLMT RC | 35 | 28 | 7.49+6.21 | 10.64+4.54 | -2.145 | **0.032** |
|  |  |  |  |  |  |  |
| DCS-II 1-5 | 35 | 28 | 17.29+11.22 | 15.82+8.58 | -0.429 | 0.668 |
| DCS 6 | 33 | 27 | 4.42+2.83 | 4.04+2.41 | -0.697 | 0.486 |
| DCS RC | 34 | 28 | 25.94+3.25 | 24.54+3.40 | -1.649 | 0.099 |
|  |  |  |  |  |  |  |
| Visuoconstruction | 34 | 28 | 29.82+10.93 | 31.00+12.60 | -0.397 | 0.691 |
| Labyrinth | 32 | 26 | 13.94+6.95 | 14.37+6.67 | -0.172 | 0.863 |
| LPS-7 | 35 | 28 | 18.26+7.21 | 17.93+9.75 | -0.630 | 0.529 |
| BDI-II | 34 | 27 | 5.62+6.51 | 7.04+7.11 | -0.542 | 0.588 |
| HAWIE-R= Hamburg Wechsler Intelligence Test – Revised version, TMT=Trail Making Test, D2=test of attention, VLMT=Verbal Learning and Memory Test, DCS=Learning and Memory Test for detecting memory deficits resulting from acquired brain damage, LPS=Performance Testing System, BDI=Beck Depression Inventory  The values are given in means and standard deviation.  VLMT: total learning (Trials 1–5), delayed free recall (Trial 7) and recognition (RC)  DCS-II: total learning (Trials 1–5), delayed free recall (Trial 6) and recognition (RC) | | | | | | |


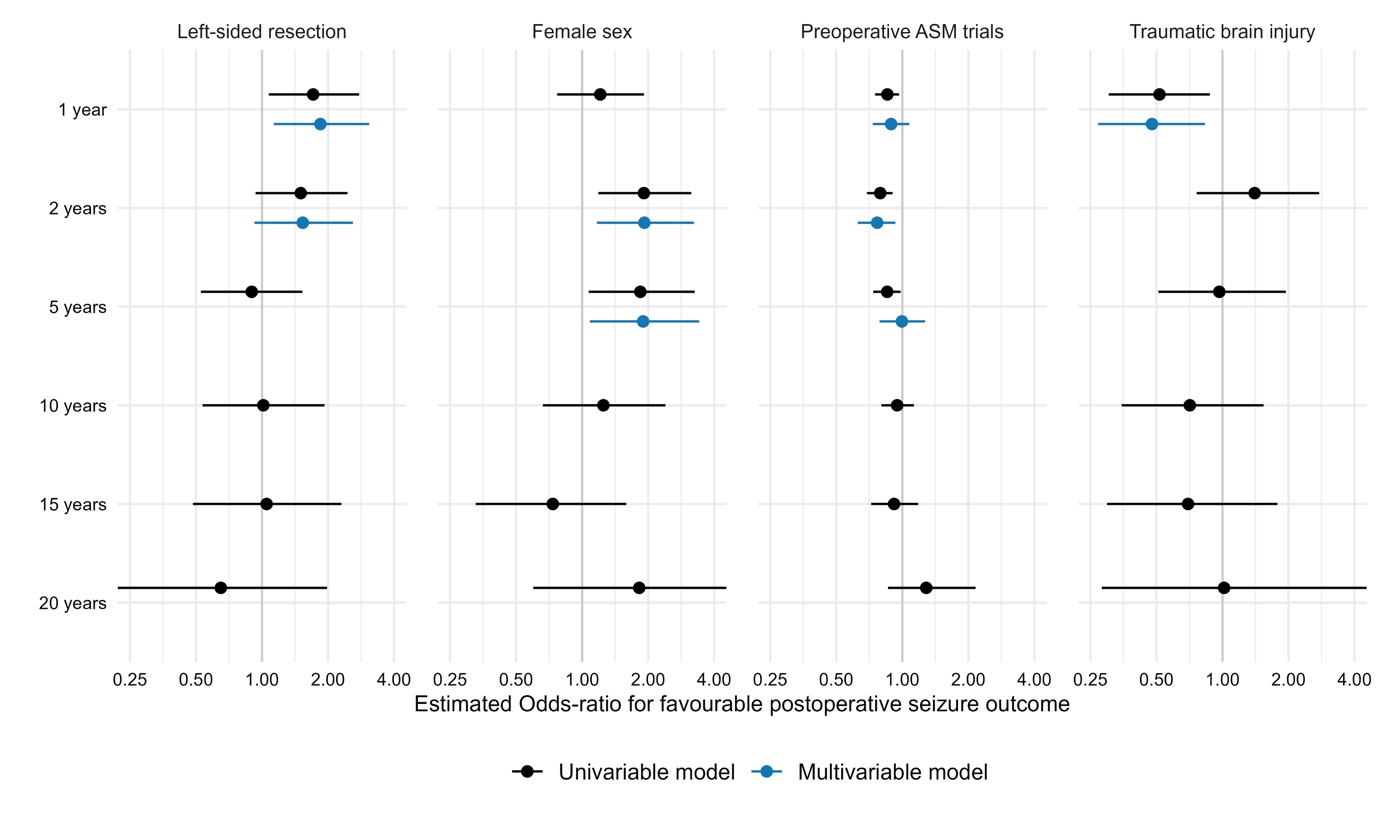


**Supplemental Fig. S1** Odds-ratios and 95% confidence intervals for favorable postoperative seizure outcome estimated from univariable and multivariable logistic regression. Separate models were estimated for each follow-up time. Confidence intervals excluding the value 1 suggest a statistically significant effect (p < 0.05)


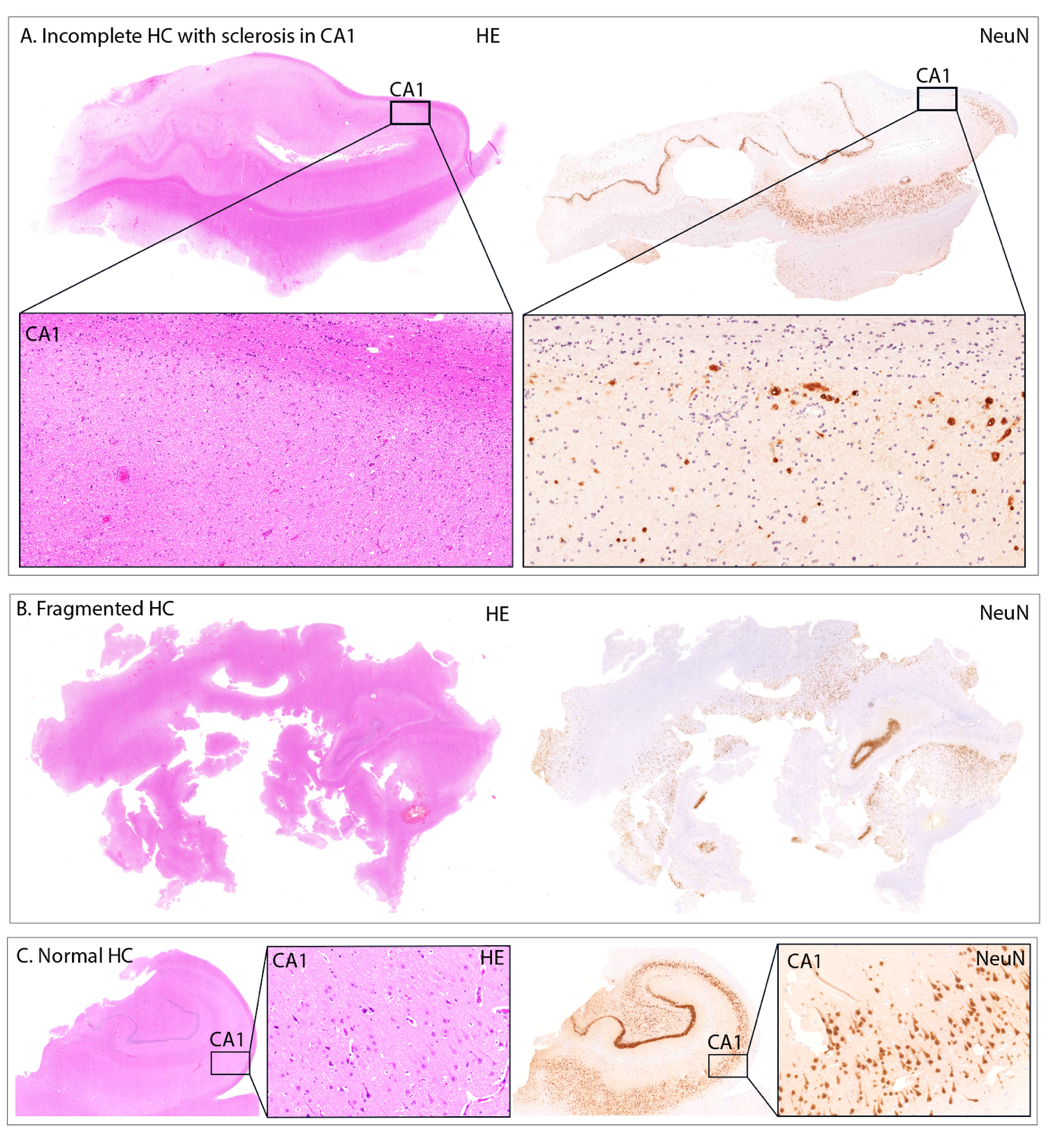


**Supplemental Fig. S2** Examples of incomplete or fragmented hippocampal samples (A & B) with normal hippocampus for reference at the bottom (C)

The ILAE requires the identification of all hippocampal sectors CA1 to CA4 for proper classification of the HS subtype. The segmental sclerosis of the CA1 sector (A) cannot be classified into a specific subtype, as not all sectors are represented in the sample for histological assessment. Fragmentation of hippocampal tissue (B) may also lead to the incapability of further classification of focal sclerosis; HC=hippocampus, HE=hematoxilina-eosina, CA=Cornu ammonis, NeuN=Anti Neuronal-Nuclei

**Supplemental References for Neuropsychological Tests**

1. Schwarzkopf-Streit C. Die Schätzung der Gesamtintelligenz aus Testkurzformen im Intelligenzkonzept nach Wechsler. . Hannover, Germany: Medical School Hannover; 2000.

2. Lux S, Helmstaedter C, Elger C. Normative study on the "Verbaler Lern- und Merkfahigkeitstest" (VLMT). Diagnostica 1999;45:7.

3. Helmstaedter C, Lendt M, Lux S. VLMT Verbaler Lern- und Merkfähigkeitstest. Göttingen: Beltz Test GmbH; 2001.

4. Helmstaedter C, Pohl C, Hufnagel A, Elger CE. Visual learning deficits in nonresected patients with right temporal lobe epilepsy. Cortex. 1991;27(4):547-55.

5. Benton A, Hamsher K. Multilingual aphasia examination. Iowa City: AJA Associates; 1989.

6. Spreen O, Strauss E. A compendium of neuropsychological tests. NewYork: Oxford University Press; 1998.

7. Brickenkamp R. Test d2 Aufmerksamkeits-Belastungs-Test. CJ H, editor. Göttingen: Verlag für Psychologie; 1968.

8. Capuis F. Labyrinth-Test LT. Bern1959.

9. Horn W. L-P-S Leistungsprüfsystem. Göttingen Hogrefe; 1983.

10. Hautzinger M, Keller F, Kühner C. Das Beck Depressionsinventar II. Deutsche Bearbeitung und Handbuch zum BDI II. Frankfurt a. M.: Harcourt Test Services; 2006.
